# Supplementary material for: Honeydew Deposition by the Giant Willow Aphid (Tuberolachnus salignus) Affects Soil Biota and Soil Biochemical Properties
Source: Insects. 2020 Jul 22;11(8):460. doi: 10.3390/insects11080460 (PMC7469182; doi:10.3390/insects11080460)
Supplement: Supplementary file 1 [file insects-11-00460-s001.pdf]

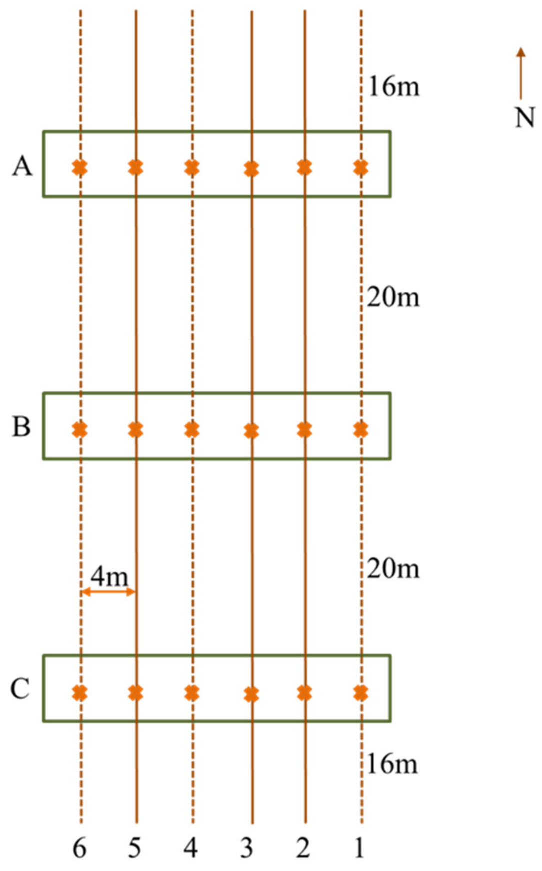

Figure S1. Sampling layout for the soil samples. The numbers (1-6) represent the rows of willow plants, and the letters (A-C) indicate the sampling points along the rows. The willow plants in rows 1, 4 and 6 were kept free of aphids, by weekly manual removal or insecticide spraying, while plants in rows 2, 3 and 5 were inoculated with aphids.

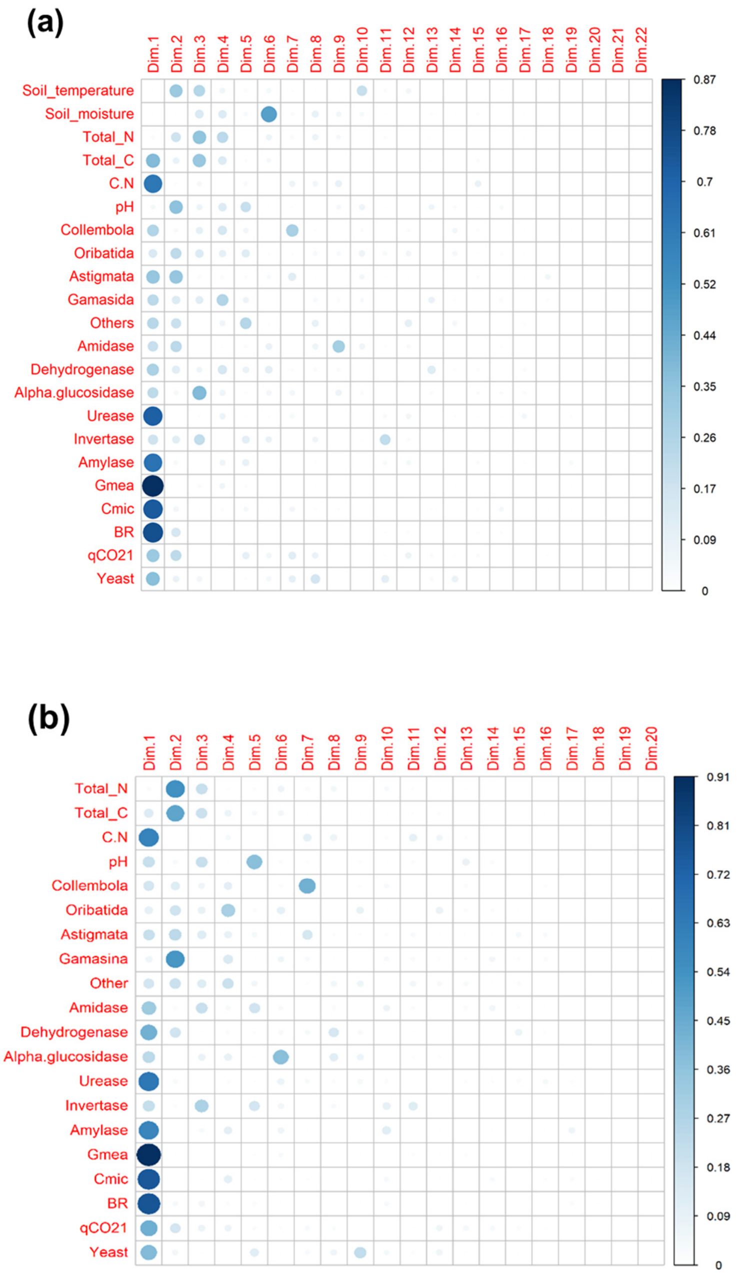

Figure S2. Correlation plots for contributing variables to each dimension in the PCA for (a) treatments, and (b) sampling times. The variables that contributed the most to the first few dimensions were selected for SEM.

Table S1. Summary of treatment effects of *T. salignus* honeydew deposition on soil biochemical properties, soil enzymes, and soil meso-fauna abundance in first and second year of the experiment (Generalized Linear Models, α=0.05).

Parameter	Unit	First year			Second year		
		df1, df2	F-value	*P-value*	df1, df2	F-value	*P-value*
pH	-	2,20	0.75	*0.485*	2,23	0.03	*0.973*
Total C	%	2,20	3.55	***0.043***	2,23	3.62	***0.044***
Total N	%	2,20	3.26	*0.061*	2,23	0.10	*0.904*
C : N	-	2,20	18.10	***0.001***	2,23	7.12	***0.004***
Collembola	ind. m^{-2}	2,20	3.27	***0.032***	2,23	3.79	***0.039***
Gamasida	ind. m^{-2}	2,20	11.45	***0.001***	2,23	0.15	*0.858*
Astigmata	ind. m^{-2}	2,20	13.49	***0.001***	2,23	4.14	***0.030***
Oribatida	ind. m^{-2}	2,20	0.82	*0.311*	2,23	3.97	***0.043***
Other mesofauna	ind. m^{-2}	2,20	3.01	***0.045***	2,23	5.97	***0.011***
Dehydrogenase	mg TPF g^{-1} soil 24 h^{-1}	2,20	3.64	***0.042***	2,23	10.25	***0.001***
Urease	NH_4^+-N g^{-1} soil 4 h^{-1}	2,20	17.29	***0.001***	2,23	20.70	***0.001***
Amidase	mg NH_4^+-N g^{-1} soil h^{-1}	2,20	3.62	***0.047***	2,23	0.09	*0.915*
β-amylase	mg starch g^{-1} soil 24 h^{-1}	2,20	34.14	***0.001***	2,23	27.03	***0.001***
Invertase	mg glucose g^{-1} soil 24 h^{-1}	2,20	21.86	***0.001***	2,23	0.26	*0.821*
α-glucosidase	mg glucose g^{-1} soil h^{-1}	2,20	4.87	***0.020***	2,23	3.90	***0.036***
Gmea	-	2,20	50.37	***0.001***	2,23	23.99	***0.001***
Cmic	µg C g^{-1} soil	2,20	44.14	***0.001***	2,23	19.90	***0.001***
BR	µg CO_2–C g^{-1} soil h^{-1}	2,20	89.54	***0.001***	2,23	45.53	***0.001***
qCO_2	µg CO_2–C µg^{-1} Cmic h^{-1}	2,20	1.51	*0.247*	2,23	28.34	***0.001***
Yeast CFU	log CFU g^{-1} soil	2,62	70.99	***0.001***	2,71	44.952	***0.001***
